# Circulating Tumor DNA (ctDNA) Clearance After Immune Checkpoint Inhibition (ICI) with Radiotherapy (RT) as a Prognostic Biomarker for Advanced Melanoma

**DOI:** 10.3390/cancers18183032

**Published:** 2026-09-18

**Authors:** Alyssa K. Steimle, Amir M. Forati, Alice Y. Zhou, Trevor McCracken, Meena M. Hosny, Golbarg Rahimi, Janmesh D. Patel, Caroline Burkey, Jessica Caraway, Taylor Huppe, Andrew Wood, Madison S. Harris, Alexander Birbrair, Jose M. Ayuso, Deepak M. Sahasrabudhe, Fauzia Hollnagel, Gary C. Doolittle, Adam R. Burr, Nina S. Mathew, Gino K. In, Adrienne Victor, Vincent T. Ma

**Affiliations:** 1 Department of Medicine, University of Wisconsin-Madison, Madison, WI 53706, USA; aforati@medicine.wisc.edu (A.M.F.); cburkey@wisc.edu (C.B.); vtma@medicine.wisc.edu (V.T.M.); 2 Department of Medicine, Division of Oncology, Washington University, St Louis, MO 63110, USA; 3 Division of Clinical Oncology, University of Kansas Medical Center, Kansas City, KS 66160, USAjcaraway@kumc.edu (J.C.); thuppe@kumc.edu (T.H.); a216w251@kumc.edu (A.W.); gdoolitt@kumc.edu (G.C.D.); nmathew3@kumc.edu (N.S.M.); 4 Department of Medicine, Division of Hematology/Oncology, University of Rochester, Rochester, NY 14642, USA; meena_hosny@urmc.rochester.edu (M.M.H.); deepak_sahasrabudhe@urmc.rochester.edu (D.M.S.); adrienne_victor@urmc.rochester.edu (A.V.); 5 Norris Comprehensive Cancer Center, University of Southern California, Los Angeles, CA 90089, USAgino.in@med.usc.edu (G.K.I.); 6 School of Medicine and Public Health, University of Wisconsin-Madison, Madison, WI 53706, USA; 7 Department of Dermatology, University of Wisconsin-Madison, Madison, WI 53706, USA; abirbrair@dermatology.wisc.edu (A.B.); jayuso@dermatology.wisc.edu (J.M.A.); 8 Department of Human Oncology, University of Wisconsin-Madison, Madison, WI 53706, USA; 9 Department of Medicine, Division of Hematology, Medical Oncology, and Palliative Care, University of Wisconsin-Madison, Madison, WI 53706, USA

**Keywords:** ctDNA, melanoma, radiation, checkpoint inhibition

## Abstract

Background/Objectives: ctDNA is evolving as an important biomarker for prognostic assessment in advanced melanoma. However, the utility of ctDNA monitoring during concurrent ICI with RT remains incompletely explored. Methods: In this multicenter retrospective study, patients with unresectable stage III/IV melanoma treated with ICI and palliative RT were identified. Patients had retrospectively collected, tumor-informed, exome-based, ctDNA monitoring within 3 months following RT. Results: A total of 50 patients treated with ICI and palliative RT were analyzed. Compared with ctDNA clearance, ctDNA detectable and increasing was associated with worse OS (adjusted HR 2.52, 95% CI 1.20–5.29; *p* = 0.015), whereas ctDNA detectable and decreasing was not significantly different from ctDNA clearance (adjusted HR 1.58, 95% CI 0.69–3.65; *p* = 0.282). One-year OS was 87.5% with persistently undetectable ctDNA, 83.3% for ctDNA clearance, 46.3% for detectable and decreasing ctDNA, and 21.4% for detectable and increasing ctDNA. In a multivariable analysis, detectable and increasing ctDNA was associated with worse OS compared with ctDNA clearance (HR 2.52, 95% CI, 1.20–5.29, *p* = 0.015). Conclusions: Among patients with advanced melanoma treated with ICI and palliative RT, ctDNA clearance is associated with significantly improved OS compared to patients with detectable and increasing ctDNA within 3 months following RT. Ongoing prospective studies are needed to understand the role of ctDNA in this population.

## Simple Summary

Circulating tumor DNA (ctDNA) can prognosticate survival outcomes in melanoma following checkpoint inhibition, but its role in advanced melanoma treated with radiation remains unclear. In this retrospective analysis of 50 patients with advanced-stage melanoma treated with checkpoint inhibition and palliative radiation, patients with clearance of ctDNA after radiation lived significantly longer compared to those with persistently detectable and increasing ctDNA. These findings suggest ctDNA monitoring could serve as a promising prognostic candidate to help stratify survival outcomes in this patient population. However, further prospective studies are needed before ctDNA monitoring can be routinely used to guide clinical decision making, treatment adaptation, or surveillance strategies in this specific group of melanoma patients.

## 1. Introduction

Although incidence of melanoma remains high, survival rates have dramatically increased in recent years [1]. This improvement in survival outcomes is largely attributed to the development and implementation of treatment with immune checkpoint inhibition [2,3]. Anti-PD-1-based therapy is now established as the frontline treatment of unresectable or metastatic melanoma [4,5,6]. Radiation therapy (RT) can also be incorporated into the treatment of metastatic melanoma with definitive or palliative intent, most often in patients with oligometastatic disease or brain metastases. Although melanoma is typically radioresistant, RT can be used in the adjuvant or palliative setting but has not been shown to improve survival outcomes [7,8]. Despite advances in systemic therapy leading to better outcomes, mortality rates for patients with metastatic and unresectable stage III/IV melanoma remain high [1,2,9,10].

Circulating tumor DNA (ctDNA) is made up of tumor-derived fragmented DNA molecules shed by malignant cells into plasma. ctDNA dynamics have been shown to have prognostic value among patients with metastatic melanoma treated with ICI. Decreasing ctDNA is associated with improved overall survival (OS), progression-free survival (PFS), and odds of disease control [11]. In another cohort of patients with metastatic melanoma, all patients with ctDNA clearance were progression-free at a median follow-up of 14.7 months, while patients with detectable ctDNA were more likely to have disease progression [12]. ctDNA has also been shown to differentiate pseudo-progression from true progression [13,14]. Trends in ctDNA may be useful in multiple clinical scenarios including determining prognosis, prognosticating the likelihood of recurrence, and distinguishing pseudo-progression.

The utility of ctDNA monitoring following radiation to sites of metastatic melanoma remains incompletely explored. Here, we describe the clinical characteristics and survival outcomes of patients with advanced-stage melanoma treated with ICI and integration of palliative RT based on ctDNA dynamics.

## 2. Methods

Patients with unresectable stage III/IV melanoma treated with ICI and palliative radiation therapy between October 2021 and June 2025 at five institutions were identified via retrospective chart review. Patients with uveal melanoma were excluded. Palliative radiation was defined as radiation to metastatic melanoma with the primary goal of improving symptom management. RT sites were collected as lesion-level data, and patients could have multiple sites of RT. There were no restrictions on site of disease or modality of radiation. Data was extracted from the electronic medical record and stored in a REDCap database. All patients were monitored with longitudinal, tumor-informed, exome-based ctDNA testing (Signatera), following the same collection and processing procedures at all institutions. ctDNA was measured in mean tumor molecules per milliliter of plasma (MTM/mL). Values below quantification limits were included. Those with a baseline ctDNA level within 0 to 90 days prior to incorporating radiation and follow-up ctDNA testing obtained within 90 days following RT were included in this analysis.

To evaluate the prognostic impact of circulating tumor DNA (ctDNA) dynamics, patients were classified according to baseline ctDNA and the most recent available post-RT ctDNA measurement within 90 days of RT initiation. Patients with undetectable ctDNA at baseline and at the qualifying follow-up measurement were classified as ctDNA persistently undetectable (baseline ctDNA 0 MTM/mL and follow-up ctDNA 0 MTM/mL). Patients with detectable ctDNA that became undetectable were classified as having ctDNA clearance. Among patients with detectable ctDNA at follow-up, values lower than baseline were categorized as ctDNA detectable and decreasing, and values greater than baseline were categorized as ctDNA detectable and increasing. Patients who had not died were censored at their recorded last known follow-up/censor date.

Multivariable Cox proportional hazards regression was utilized to estimate the association between ctDNA trends and overall survival, adjusting for predefined clinical covariates including age (≥65 vs. <65 years), disease stage (IV vs. III), brain or leptomeningeal radiation (yes vs. no), and melanoma subtype (cutaneous vs. other). All hazard ratios for the multivariable analysis were reported relative to the ctDNA clearance as the reference group, and all hazard ratios were adjusted HR. The overall survival time origin was the radiation start date. To account for the limited sample size relative to the number of covariates, which introduced numerical instability and complete separation in standard unpenalized models, a penalized Cox model (Ridge/L2 regularization) was employed as the primary analytical strategy. All covariates in the primary model were entered as binary indicator variables coded 0/1; no additional user-applied scaling was performed. Model fitting used the default lifelines convergence criteria. All eight candidate ridge penalties completed all five cross-validation folds without convergence warnings or failures, and the final λ = 0.25 model converged successfully. The ridge penalty parameter (λ) was selected from a candidate grid of 0.01–2.0 using 5-fold stratified cross-validation, with λ = 0.25 selected by maximizing mean held-out log-likelihood. Hazard ratios were estimated from the penalized partial-likelihood fit, with model-based Wald 95% confidence intervals and *p*-values. The effective degree of freedom (EDF) of the final model was estimated to be 3.91. Sensitivity analyses included the full unpenalized model, a ridge-penalized model with a fixed λ = 0.50, and a parsimonious ctDNA-only model.

To address potential look-ahead and immortal-time bias in post-RT ctDNA classification, two complementary analyses were performed. A 90-day landmark analysis included patients alive and under observation at 90 days with an evaluable ctDNA measurement. ctDNA status was defined by the most recent measurement within 90 days of RT initiation, and OS was measured from the landmark. A time-dependent Cox model measured OS from RT initiation, with ctDNA status updated at each measurement. Ridge-penalized Cox regression was used due to separation in the unpenalized model. The penalty parameter was selected using patient-level cross-validation to prevent intervals from the same patient from occurring in both training and validation sets, and λ = 0.10 was selected.

Among patients with detectable baseline ctDNA, molecular response was defined as

$$MR_{c}={log}_{10}\left(B+c\right)-{log}_{10}\left(F+c\right)={log}_{10}\left(\frac{B+c}{F+c}\right),$$

where *B* is baseline ctDNA, *F* is the most recent qualifying follow-up ctDNA measurement within 90 days, and *c* is a stabilizing constant. A primary value of *c* = 0.01 MTM/mL was used because it was below the minimum observed positive ctDNA concentration (0.03 MTM/mL), allowing zero values to be log-transformed while minimizing perturbation of positive measurements. Sensitivity analyses were performed using *c* = 0.003 and *c* = 0.03 MTM/mL. All statistical analyses and visualizations were performed using Python version 3.13.15, utilizing the lifelines version 0.30.3 for survival modeling.

## 3. Results

**Figure 1:**
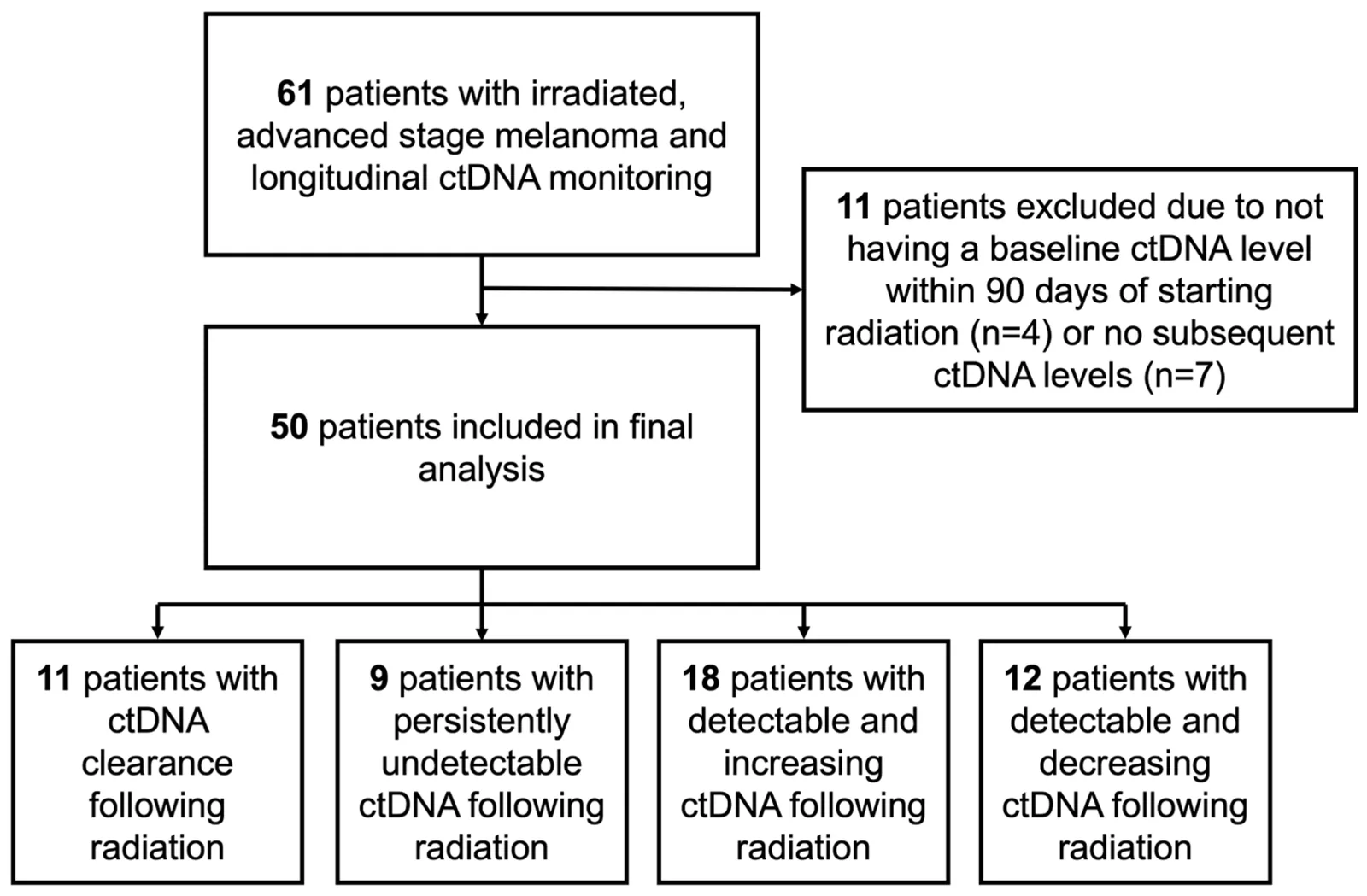
Study flow diagram describing the inclusion criteria and final number of patients included in each group based on ctDNA trends.

Sixty-one patients with irradiated, advanced-stage melanoma and longitudinal ctDNA monitoring were identified. Of these 61 patients, 4 did not have a baseline ctDNA level within 90 days prior to initiation of RT, and 7 patients did not have follow-up ctDNA testing drawn within 90 days after RT. These 11 patients were excluded, and the remaining 50 patients that met criteria were included in this analysis. Some 11 patients had ctDNA clearance, 12 had ctDNA detectable and decreasing, 18 had ctDNA detectable and increasing, and 9 had ctDNA persistently undetectable (Figure 1).

Patient characteristics are described in Table 1. Median age was 65 (24–86), and the median baseline ctDNA level was 8.9 MTM/mL (0–11,390 MTM/mL). Patients had a median of 3 ctDNA draws (range of 2–5) included from baseline to follow-up. Baseline ctDNA was collected a median of 17.5 days before RT initiation (with a range of 77 days before to 6 days after RT initiation). ctDNA follow-up draws were collected at a median of 28 day intervals (4–105 days) following RT. Observed median follow-up was 7.1 months, and reverse-KM median was 12.2 months from RT start date. Primary sites of melanoma included 41 patients with cutaneous melanoma (82%), 5 with mucosal (10%), and 4 patients with melanoma of unknown primary (8%). The majority (92%) of patients had stage IV disease, including 5 (10.0%) each with M1a and M1b stage disease, 12 (24.0%) with M1c, and 24 patients (48.0%) with M1d staging. Three patients (6.0%) had stage IIIC melanoma, and 1 (2.0%) had stage IIID disease. ICI regimens included 70% (*n* = 35) anti-PD-1/anti-CTLA-4, 16% (*n* = 8) anti-PD-1/anti-LAG-3, 2% (*n* = 1) anti-PD-1/investigational ICI administered within a clinical trial, and 12% (*n* = 6) anti-PD-1 monotherapy. A total of 13 (26.0%) patients had surgery while receiving checkpoint inhibitor therapy. The most common RT sites were the central nervous system (50%, *n* = 25) and skin/soft tissue (24%, *n* = 12), followed by lymph nodes and bone (each 14%, *n* = 7), and lung and liver (each 6%, n = 3).

**Figure 2:**
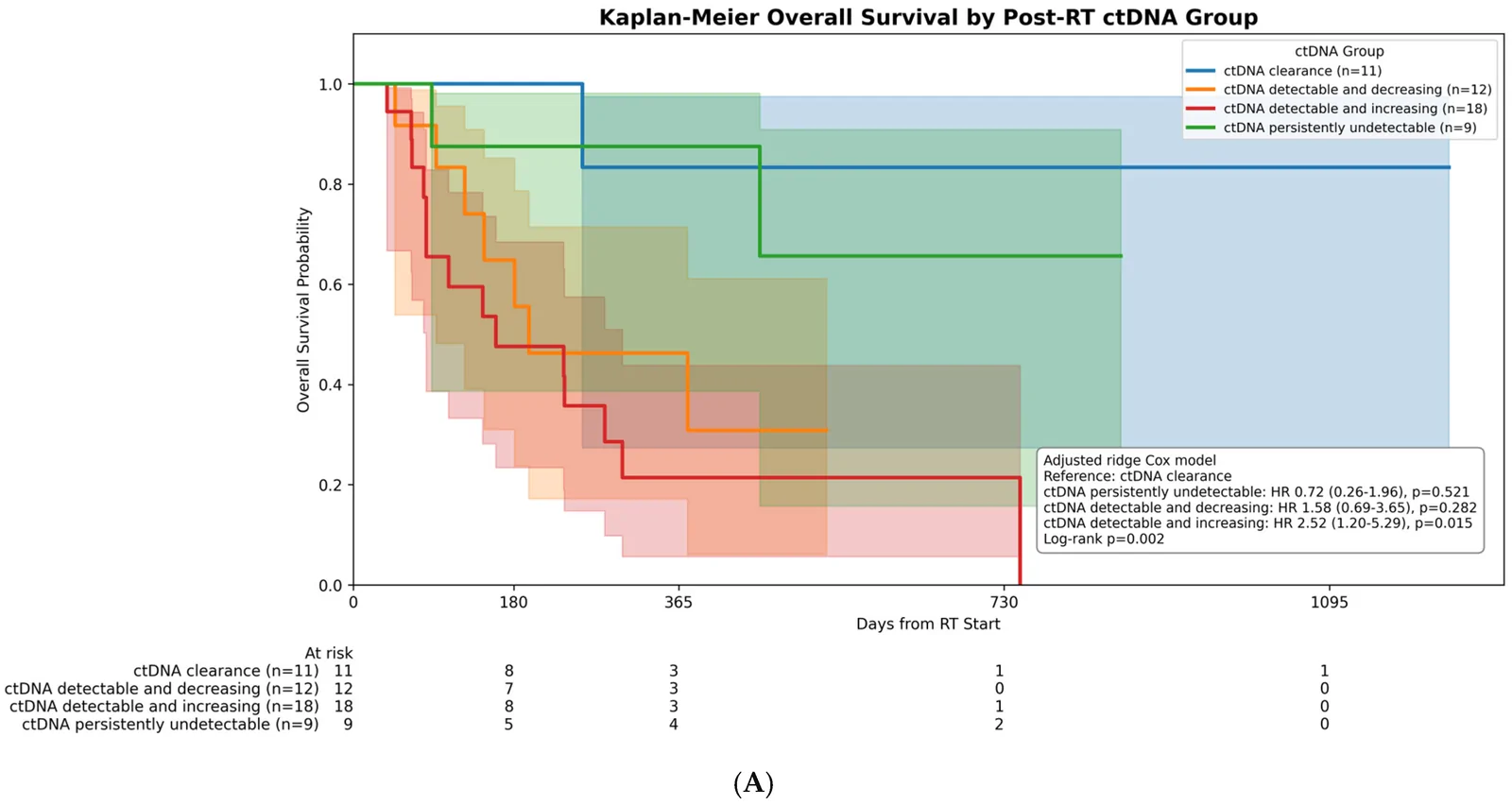

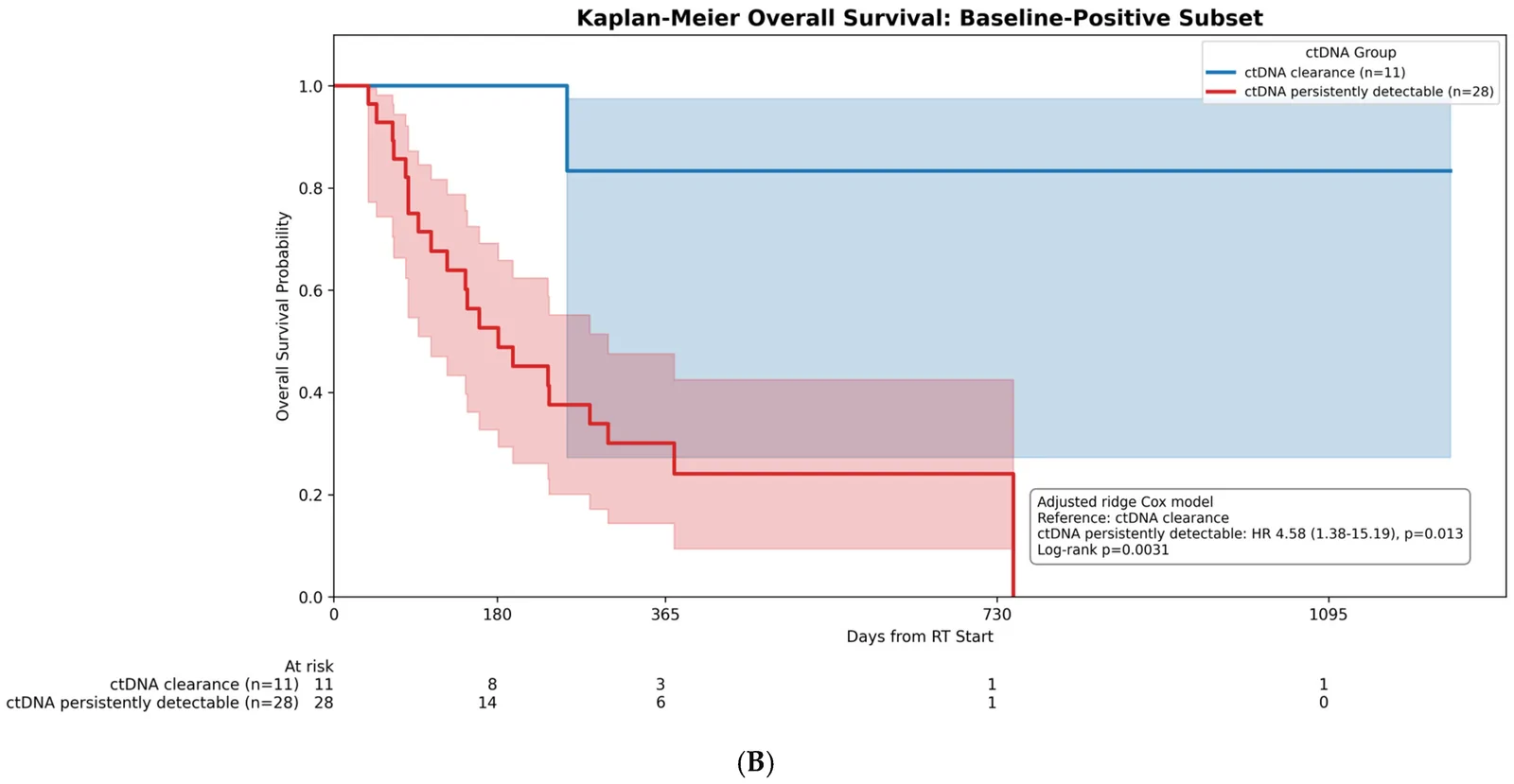
Kaplan–Meier plots for overall survival (OS) for patients with advanced, irradiated melanoma and ctDNA clearance compared to detectable and increasing ctDNA levels, detectable and decreasing ctDNA levels, and persistently undetectable ctDNA (**A**). Kaplan–Meier plots for OS comparing patients with ctDNA clearance to persistently detectable ctDNA (**B**). The index date for both Kaplan–Meier plots is the start date of radiation.

Overall survival was significantly longer among patients with clearance of ctDNA compared to those with increasing ctDNA levels following RT. There were 24 deaths and 26 censored over a median follow-up of 7.1 months (interquartile range 3.3–12.0 months), with a reverse Kaplan Meier median follow-up of 12.2 months. Total person time was 38.7 patient-years. One-year overall survival was 83.3% (95% CI 27.3–97.5%) among patients with ctDNA clearance, 87.5% (95% CI 38.7–98.1%) for those with persistently undetectable ctDNA, 46.3% (95% CI 17.2–71.4%) for those with decreasing ctDNA, and 21.4% (95% CI 5.7–43.8%) for those with increasing ctDNA (Figure 2A). Compared to clearance of ctDNA within 90 days following RT, increasing ctDNA levels were associated with worse overall survival, with a hazard ratio of 2.52 (*p* = 0.015, 95% CI 1.20–5.29) (Table 2). The cohorts with decreasing or persistently undetectable ctDNA concentrations did not have significantly different overall survival than those with ctDNA clearance, with a hazard ratio of 1.58 (0.282, 95% CI 0.69–3.65) and 0.72 (0.521, 95% CI 0.26–1.96), respectively. Comparing ctDNA clearance or persistently undetectable ctDNA with all patients with detectable ctDNA, including those with increasing and decreasing ctDNA levels, resulted in a statistically significant difference in overall survival. Among patients with detectable baseline ctDNA, persistently detectable ctDNA was associated with worse OS compared with true clearance (adjusted HR 4.58, 95% CI 1.38–15.19, *p* = 0.013 (Figure 2B). Median OS was not reached in the ctDNA clearance or ctDNA persistently undetectable groups. Median OS was 6.5 months in the ctDNA detectable and decreasing group and 5.3 months in the ctDNA detectable and increasing group.

The 90-day landmark analysis included 39 of the 50 patients. A total of 7 patients had persistently undetectable ctDNA, 10 had ctDNA clearance, and 11 each had increasing and decreasing ctDNA levels. Using ctDNA clearance as the reference group, ctDNA detectable and increasing was associated with a higher hazard of death (HR 8.09, 95% CI 1.01–64.80; *p* = 0.048). ctDNA detectable and decreasing (HR 7.38, 95% CI 0.88–61.88; *p* = 0.065) and ctDNA persistently undetectable (HR 1.07, 95% CI 0.07–17.33; *p* = 0.961) were not significantly different from ctDNA clearance.

The time-dependent ctDNA Cox analysis included all 50 patients with 161 intervals and 24 deaths. Currently detectable ctDNA was independently associated with worse overall survival (HR 2.62, 95% CI 1.28–5.37, *p* = 0.009) when adjusting for age, site of radiation, stage, and primary melanoma type. Sensitivity analysis resulted in worse overall survival (HR 1.79, 95% CI 1.40–2.29, *p* < 0.001) per 10-fold increase in ctDNA.

Among the 31 patients with detectable baseline ctDNA who were eligible for the 90-day landmark analysis, greater log10 molecular response showed a directionally favorable but non-significant association with OS. Using the primary stabilizing constant *c* = 0.01 MTM/mL, each 1-unit increase in log10 molecular response was associated with an HR of 0.83 (95% CI 0.63–1.08; *p* = 0.161). Results were similar using *c* = 0.003 MTM/mL (HR 0.82, 95% CI 0.64–1.05; *p* = 0.124) and *c* = 0.03 MTM/mL (HR 0.83, 95% CI 0.63–1.11; *p* = 0.208), indicating that the interpretation was not materially affected by the choice of stabilizing constant.

**Figure 3:**
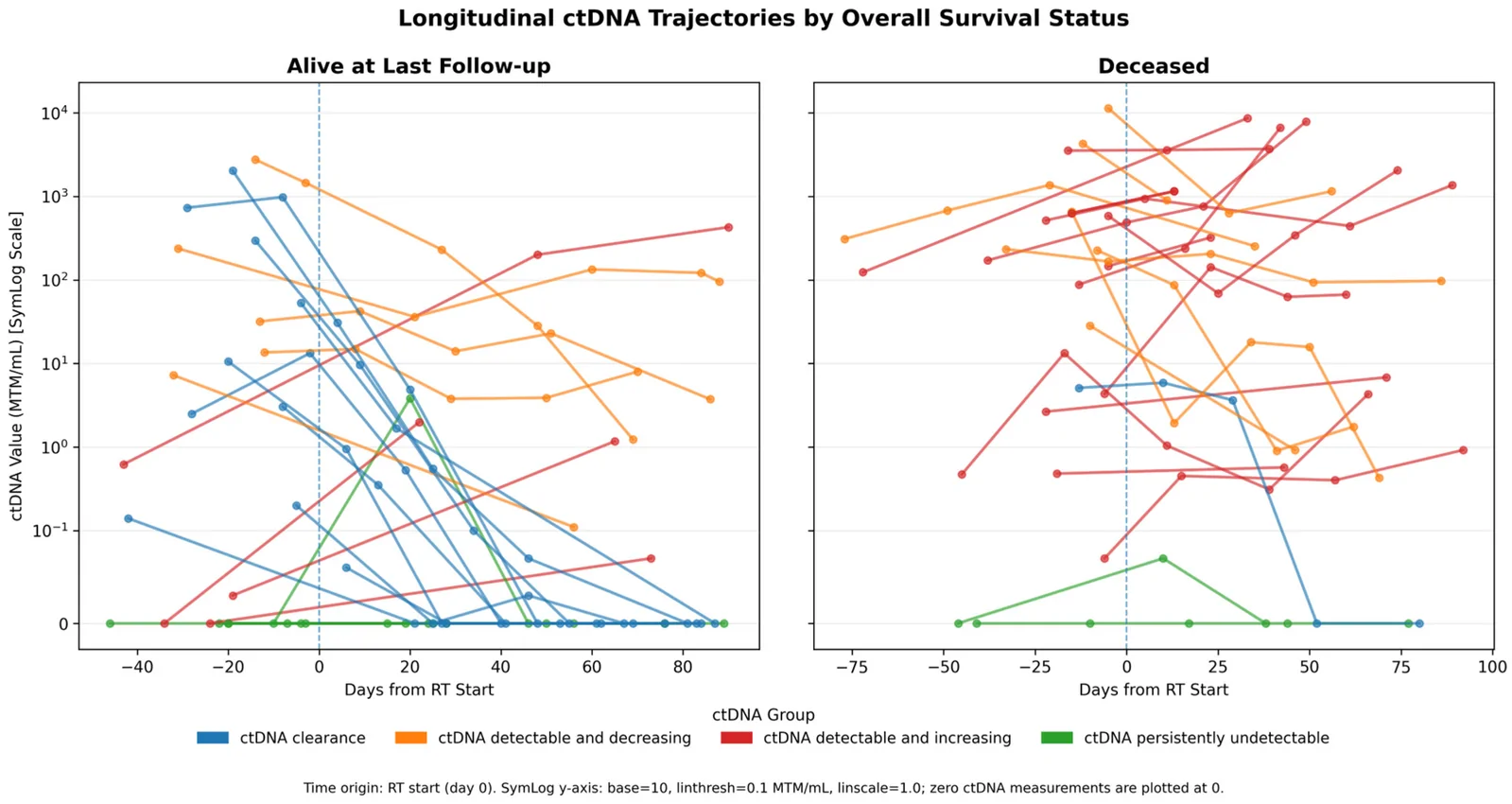
Spider plots of each patient’s ctDNA levels obtained at the treating physician’s discretion based on survival at follow-up.

Patients with clearance of ctDNA were more likely to be alive at the time of follow-up, and those with increasing or decreasing yet detectable ctDNA were more likely to be deceased. Individual patients had variable duration of follow-up and frequency of ctDNA monitoring. Additionally, logarithmic ctDNA values were higher among deceased patients than alive (Figure 3).

In the ridge-penalized multivariate analysis, increasing ctDNA was independently associated with worse OS compared with ctDNA clearance (HR of death 2.52, 95% CI 1.20–5.29, *p* = 0.015). Decreasing ctDNA (HR 1.58, 95% CI 0.69–3.65, *p* = 0.282) and baseline 0 → 0 (HR 0.72, 95% CI 0.26–1.96, *p* = 0.521) were not statistically significantly different from clearance. A total of 50 patients were included in the ridge-penalized model, with 24 deaths and 26 censored observations. There was no statistically significant difference between patients with age greater than 64 versus patients 64 and younger (*p* = 0.636, HR 1.17, 95% CI 0.60–2.29). There was also no significant difference between those with radiation to the brain or leptomeninges compared to patients who received radiation to other sites of disease (*p* = 0.800, HR 1.09, 95% CI 0.56–2.13). Differences between stage IV and stage III disease were insignificant (*p* = 0.419, HR 1.94, 95% CI 0.39–9.63), as were differences between cutaneous melanoma and mucosal or unknown primary site of melanoma (*p* = 0.986, HR 1.01, 95% CI 0.42–2.43) (Table 2).

## 4. Discussion

Circulating tumor DNA has emerged as a robust prognostic indicator in patients with melanoma. Detectable ctDNA levels both before and after any systemic therapy have been associated with diminished progression-free and overall survival [15,16,17]. In patients receiving checkpoint inhibitors, persistently detectable ctDNA following treatment initiation similarly correlates with worse PFS and OS [18]. Similarly, early ctDNA dynamics within only 3–4 weeks after starting ICI therapy have proven to be predictive of both OS and PFS as well [11]. By contrast, less is understood about the prognostic implications of ctDNA following radiation in patients with advanced-stage melanoma. In this retrospective cohort, ctDNA clearance was associated with significantly improved OS compared with detectable and increasing ctDNA levels within 3 months following initiation of RT. This finding identifies post-radiation ctDNA status as a promising prognostic candidate that warrants prospective evaluation.

These results establish a prognostic association which should be considered when using ctDNA monitoring in clinical practice for patients with advanced melanoma following treatment with RT. Serial ctDNA assessment within 3 months after initiating radiation may be an accessible and informative tool to guide prognostication. Patients with continually detectable ctDNA represent a clinically distinct, higher-risk population who may be particularly well suited to enrollment in prospective clinical trials investigating treatment adaptation based on ctDNA dynamics. This approach could potentially improve both overall and progression-free survival in this group of patients with especially poor prognosis.

The use of penalized regression models (Ridge/L2 regularization) in our survival analysis is highly justified given our sample size (n = 50) relative to the number of covariates which introduced complete separation and numerical instability in standard unpenalized Cox models. The methodological literature demonstrates that regularized modeling is a critical strategy for biomarker discrimination and model evaluation in high-dimensional or low-sample-size settings, providing stable parameter estimates where classical methods fail [19]. Furthermore, the identification of a prognostic molecular marker in a retrospective cohort must be distinguished from the validation of a patient-level clinical prediction model. Cross-disease methodological frameworks have underscored that identifying prognostic biomarkers associated with disease risk is a separate scientific task from establishing a fully validated clinical prediction tool ready for direct patient-level prediction [20].

One hypothesis to explain the observed association is that patients receiving radiation in combination with immunotherapy may represent a biologically distinct population. Radiation-induced tumor cell death can lead to immunogenic cell death, releasing tumor antigens and increasing damage-associated molecular pattern molecules (DAMPs). This upregulation of tumor antigens and DAMPs leads to potential enhancement of the immune system within the tumor microenvironment, which can prime patients for checkpoint inhibitor therapy [21,22,23,24]. Under this hypothetical framework, persistent ctDNA detection might reflect not only residual tumor burden but also an inadequate immune response. However, the present data cannot distinguish these possibilities.

A related hypothesis is that radiation may transiently increase ctDNA shedding into the systemic circulation. In other cancers, radiation has been associated with an initial increase in cell free DNA in the blood [25,26,27]. This could lead to systemic immune system exposure to tumor neoantigens. Multiple studies have demonstrated size reduction of non-irradiated metastases following local radiotherapy, especially in patients with ICI refractory disease [28,29,30,31], though clinical abscopal responses after combined RT and ICI remain heterogeneous and inconsistently reproduced. When ctDNA remains detectable despite this combined local and systemic immune activation, it may indicate tumor evasion of both radiation-induced and checkpoint inhibitor-mediated immune control, identifying a more aggressive disease biology associated with worse survival outcomes. These mechanisms are speculative in the context of this study and were not measured here. Further studies are necessary to clarify the extent to which radiation enhances ICI efficacy in patients with advanced-stage melanoma and how ctDNA dynamics might function as a biomarker in this setting.

These biological mechanisms linking radiotherapy-induced immunogenic cell death, local ctDNA shedding, systemic immune activation, and overall survival remain speculative in the context of our study. Unlike experimentally focused oncology research where specific cell-death pathways are measured and validated via direct cellular and molecular assays [32], our retrospective data cannot demonstrate these mechanisms. While this phenomenon is biologically plausible and supported by case series demonstrating abscopal effects [28,29,30,31], our clinical observations represent correlations rather than causal evidence of immune priming. This distinction between identifying a prognostic molecular marker associated with disease risk and demonstrating immediate clinical utility for treatment adaptation is critical, as established in other disease contexts [33,34]. Therefore, our findings should be interpreted as generating biological hypotheses and identifying ctDNA as a promising prognostic candidate rather than supporting immediate biomarker-guided treatment adaptation in routine clinical practice.

Several limitations inherent in this study’s retrospective, multi-institutional design warrant a cautious interpretation, including its small sample size leading to reduced power of statistical analysis, short duration of ctDNA monitoring, and variable intervals of ctDNA monitoring and follow-up duration. Its retrospective design introduces potential ascertainment and selection biases related to ctDNA testing, missing data, institutional heterogeneity, and heterogeneous RT and ICI exposures, with residual confounding remaining possible despite adjustment. Additionally, the lack of standardized imaging-response assessment may have introduced variability in outcome classification and should be considered when interpreting these findings.

We were also unable to evaluate several melanoma-specific prognostic factors, including performance status, surgical details, CNS versus extracranial disease, prior systemic therapy, and timing of ICI initiation relative to RT, as these data were not systematically collected. Likewise, data regarding radiation dose, number of fractions, and number of lesions were not collected. Several melanoma-specific clinical and genomic characteristics, such as *BRAF* mutation status, LDH levels, and tumor burden were not systematically collected. The recent literature reaffirms the importance of molecular subtype-specific melanoma biology, suggesting that omitting genomic and molecular characteristics like *BRAF* status can confound prognostic interpretation [35]. Prognostic modeling and tumor stratification can account for this complex biological and clinical heterogeneity [36]. Our cohort presents substantial treatment heterogeneity, including diverse ICI regimens and radiation exposures. Treating all immunotherapy-treated patients as a homogeneous group may be a simplification, as demonstrated in other clinical contexts that have reported treatment-specific exposures that are vital to isolating biological effects [37].

The bioanalytical sampling process also carries key limitations. Quantitative circulating measurements, such as ctDNA, require rigorous calibration and analytical validation—analogous to standards in other small molecule bioanalytical assays—to ensure technical reproducibility and establish precise limits of detection and quantification [38]. It is also important to recognize that ctDNA sensitivity can differ significantly depending on the site of metastatic disease, being notably lower in patients with isolated CNS disease [39]. In the multivariate analysis presented here, there was no significant difference between patients with brain or leptomeningeal radiation compared to other sites of disease. Further investigation comparing ctDNA levels in patients with radiated versus non-radiated central nervous system disease is warranted. Methodological research in other fields highlights the need to explicitly model and compensate for such measurement sensitivity, rather than assuming equivalent detectability across all clinical conditions [40].

Larger, prospective trials with longer duration of follow-up are warranted to further characterize the clinical utility of ctDNA for patients with advanced-stage melanoma after radiation therapy. In addition to overall survival analysis, calculating progression-free survival as well as objective response rates in this population would also be valuable. It would also be useful to better understand how the durability of ctDNA clearance beyond 3 months after radiation impacts survival outcomes. Trials specifically investigating treatment adaptation and adjusting imaging frequency are also necessary to determine the clinical utility of ctDNA. Future prospective studies should prospectively define ctDNA sampling time points, clinical and imaging endpoints, ctDNA-triggered management strategies, and lesion-level response assessments that distinguish irradiated from non-irradiated lesions. Furthermore, whether the degree of change in ctDNA would also impact prognostication in many patient scenarios should be clarified. Collectively, these findings identify ctDNA as a promising biomarker in melanoma necessitating further investigation in prospective studies.

## 5. Conclusions

The prognostic value of ctDNA in melanoma has been described well previously; however, its role after RT in advanced disease is less understood. Here, ctDNA clearance within 3 months of RT was associated with significantly better OS than those with increasing ctDNA. There was not a significant difference in OS between ctDNA clearance and either decreasing or persistently undetectable ctDNA within 3 months of RT. Further research with prospective analysis, longer follow-up duration, and assessment of PFS and response rates are needed to validate ctDNA as a biomarker in this setting.

## Figures and Tables

**Table 1:** Baseline characteristics.

Characteristic	All (*n* = 50)	ctDNA Persistently Undetectable (*n* = 9)	ctDNA Clearance (*n* = 11)	ctDNA Detectable and Decreasing (*n* = 12)	ctDNA Detectable and Increasing (*n* = 18)
Age—Median (Range)					
years	65 (24–86)	59 (40–78)	60 (34–75)	67 (41–76)	65 (24–86)
Melanoma Type—No. (%)					
Cutaneous	41 (82.0)	8 (88.9)	8 (72.7)	9 (75.0)	16 (88.9)
Mucosal	5 (10.0)	0 (0.0)	1 (9.1)	2 (16.7)	2 (11.1)
Unknown Primary	4 (8.0)	1 (11.1)	2 (18.2)	1 (8.3)	0 (0.0)
AJCC Stage—No. (%)					
IIIC	3 (6.0)	1 (11.1)	1 (9.1)	0 (0.0)	1 (5.6)
IIID	1 (2.0)	0 (0.0)	0 (0.0)	0 (0.0)	1 (5.6)
IV	46 (92.0)	8 (88.9)	10 (90.9)	12 (100.0)	16 (88.9)
M1a	5 (10.0)	1 (11.1)	0 (0.0)	2 (17.0)	2 (11.0)
M1b	5 (10.0)	1 (11.1)	0 (0.0)	2 (17.0)	2 (11.0)
M1c	12 (24.0)	2 (22.2)	1 (9.1)	3 (25.0)	6 (33.0)
M1d	24 (48.0)	4 (44.4)	9 (81.8)	5 (42.0)	6 (33.0)
Immunotherapy Type—No. (%)					
Ipi + Nivo	35 (70.0)	2 (22.2)	11 (100.0)	10 (83.3)	12 (66.7)
Nivo + Rela	8 (16.0)	4 (44.4)	0 (0.0)	2 (16.7)	2 (11.1)
Anti-PD-1 monotherapy	6 (12.0)	3 (33.3)	0 (0.0)	0 (0.0)	3 (16.7)
Anti-PD-1 + investigational ICI	1 (2.0)	0 (0.0)	0 (0.0)	0 (0.0)	1 (5.6)
Surgery During IO—No. (%)					
	13 (26.0)	4 (44.4)	3 (27.3)	3 (25.0)	3 (16.7)
Radiation Site—No. (%)					
Skin/Muscle	12 (24.0)	2 (22.2)	2 (18.2)	3 (25.0)	5 (27.8)
Lymph Node	7 (14.0)	2 (22.2)	0 (0.0)	1 (8.3)	4 (22.2)
Lung/Pleura	3 (6.0)	0 (0.0)	0 (0.0)	2 (16.7)	1 (5.6)
Liver	3 (6.0)	0 (0.0)	0 (0.0)	2 (16.7)	1 (5.6)
Bone	7 (14.0)	1 (11.1)	1 (9.1)	1 (8.3)	4 (22.2)
Brain	25 (50.0)	5 (55.6)	8 (72.7)	5 (41.7)	7 (38.9)
Baseline ctDNA—Median (IQR)					
MTM/mL	8.9 (0.06–281.29)	0 (0–0)	5.1 (1.35–174.30)	236.1 (31.04–1181.76)	46.24 (0.47–430.85)

AJCC: American Joint Committee on Cancer; ctDNA: circulating tumor DNA; IO: immunotherapy; Ipi: ipilimumab; IQR: interquartile range; Nivo: Nivolumab; Rela: Relatlimab.

**Table 2:** Multivariable analysis using ridge-penalized estimates for overall survival. Hazard ratios describe the hazard of death.

Variable	Hazard Ratio (HR)	95% Confidence Interval	*p*-Value
Age ≥ 65	1.17	0.60–2.29	0.636
Brain/leptomeninges RT (vs. other site of RT)	1.09	0.56–2.13	0.800
Stage IV (vs. III)	1.94	0.39–9.63	0.419
Cutaneous melanoma (vs. other)	1.01	0.42–2.43	0.986
ctDNA persistently undetectable (vs. ctDNA clearance)	0.72	0.26–1.96	0.521
ctDNA detectable and decreasing (vs. ctDNA clearance)	1.58	0.69–3.65	0.282
ctDNA detectable and increasing (vs. ctDNA clearance)	2.52	1.20–5.29	0.015 *

* *p* < 0.05 = statistically significant.

## Data Availability

The datasets presented in this article are not readily/publicly available due to time limitations, per the IRB exemption protocol. Requests to access the datasets should be directed to Dr. Vincent T. Ma.
